# Successful treatment of BK virus‐associated severe hemorrhagic cystitis with bilateral single‐J ureteral stenting

**DOI:** 10.1002/iju5.12445

**Published:** 2022-04-26

**Authors:** Akira Fujita, Kohei Kobatake, Takafumi Fukushima, Kenshiro Takemoto, Syunsuke Miyamoto, Hiroyuki Kitano, Kenichiro Ikeda, Keisuke Goto, Keisuke Hieda, Shuhei Karakawa, Tetsutaro Hayashi, Jun Teishima, Nobuyuki Hinata

**Affiliations:** ^1^ Department of Urology Hiroshima University Hospital Hiroshima Japan; ^2^ Department of Pediatrics Hiroshima University Hospital Hiroshima Japan

**Keywords:** BK virus, hematopoietic stem cell transplantation, hemorrhagic cystitis, ureteral stent, urinary diversion

## Abstract

**Introduction:**

BK virus‐associated hemorrhagic cystitis is a significant complication of hematopoietic stem cell transplantation. Although severe BK virus‐associated hemorrhagic cystitis is associated with treatment‐related mortality, sufficient evidence regarding its management is lacking.

**Case presentation:**

A 14‐year‐old boy presented with BK virus‐associated hemorrhagic cystitis and bladder clot retention after hematopoietic stem cell transplantation. Various urological interventions failed to improve cystitis. While bladder clot retention frequently recurred, surgical intervention was difficult because of the underlying hematological disorder. Hence, bilateral single‐J ureteral stenting followed by Foley catheter placement was performed as a urinary diversion. The bladder clot completely disappeared 27 days after stenting. No additional procedure was required. BK virus‐associated hemorrhagic cystitis did not recur after the blood clot disappeared.

**Conclusion:**

Bilateral single‐J ureteral stenting followed by Foley catheter placement is a simple and effective treatment method and should be considered before surgical intervention for severe BK virus‐associated hemorrhagic cystitis.

**Abbreviations & Acronyms**
BKV‐HC: BK virus‐associated hemorrhagic cystitis
CBI: continuous bladder irrigation
CRP: C‐reactive protein
Hb: hemoglobin
HC: hemorrhagic cystitis
HSCT: hematopoietic stem cell transplantation
PCy: percutaneous cystostomy
Plt: platelet
PN: percutaneous nephrostomy
RCC: red cell concentrate
TUE: transurethral electrocoagulation

**Keynote message**

The authors report the first case of BK virus‐associated severe hemorrhagic cystitis successfully treated with bilateral single‐J ureteral stenting, followed by Foley catheter placement. As the proposed method is simple and effective, it should be considered before surgical intervention for severe BKV‐HC.

## Introduction

BK viruria can lead to BKV‐HC, a well‐recognized and significant complication in up to 40% of recipients of HSCT.^1^, ^2^ Grades IV HC, which requires instrumentation for clot evacuation, accounts for 15.1% of HC after HSCT.^3^ While such severe cases are associated with treatment‐related mortality, a standard treatment for BKV‐HC has not yet been established.^4^, ^5^ Several surgical approaches^6^, ^7^, ^8^ have been reported; however, the ultimate outcome greatly depends on the general condition of the patient and treatment of the underlying hematological disorder.^9^ Herein, we report the first case of successful bilateral single‐J ureteral stenting followed by Foley catheter placement for severe BKV‐HC.

## Case presentation

**Fig. 1:**
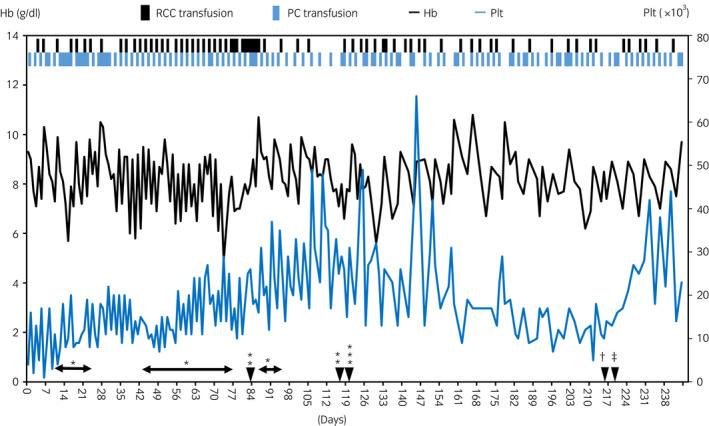
Course of BKV‐HC. Changes in the Hb and Plt counts in the peripheral blood and the time of urological intervention. *CBI; **TUE; ***bilateral single‐J ureteral stenting and Foley catheter placement; †removal of the stents; ‡removal of the catheter. [Colour figure can be viewed at wileyonlinelibrary.com]

A 14‐year‐old boy with acute lymphocytic leukemia developed slight hematuria 4 days after HSCT at our hospital. Urine tests revealed significantly increased BK virus levels of 5.0 × 10^9^ copies/mL, while adeno and JC virus levels were normal. No bacteriuria was observed. A Foley catheter was placed for the diagnosis of BKV‐HC, and urological intervention was needed as bladder retention occurred on day 12 due to a blood clot. The purchase of Cidofovir (not approved in Japan), which was reported to be effective in several reports,^10^ was postponed due to financial issues. Frequent transfusions of RCC and PCy failed to improve Hb level and Plt count after HSCT (Fig. 1).

**Fig. 2:**
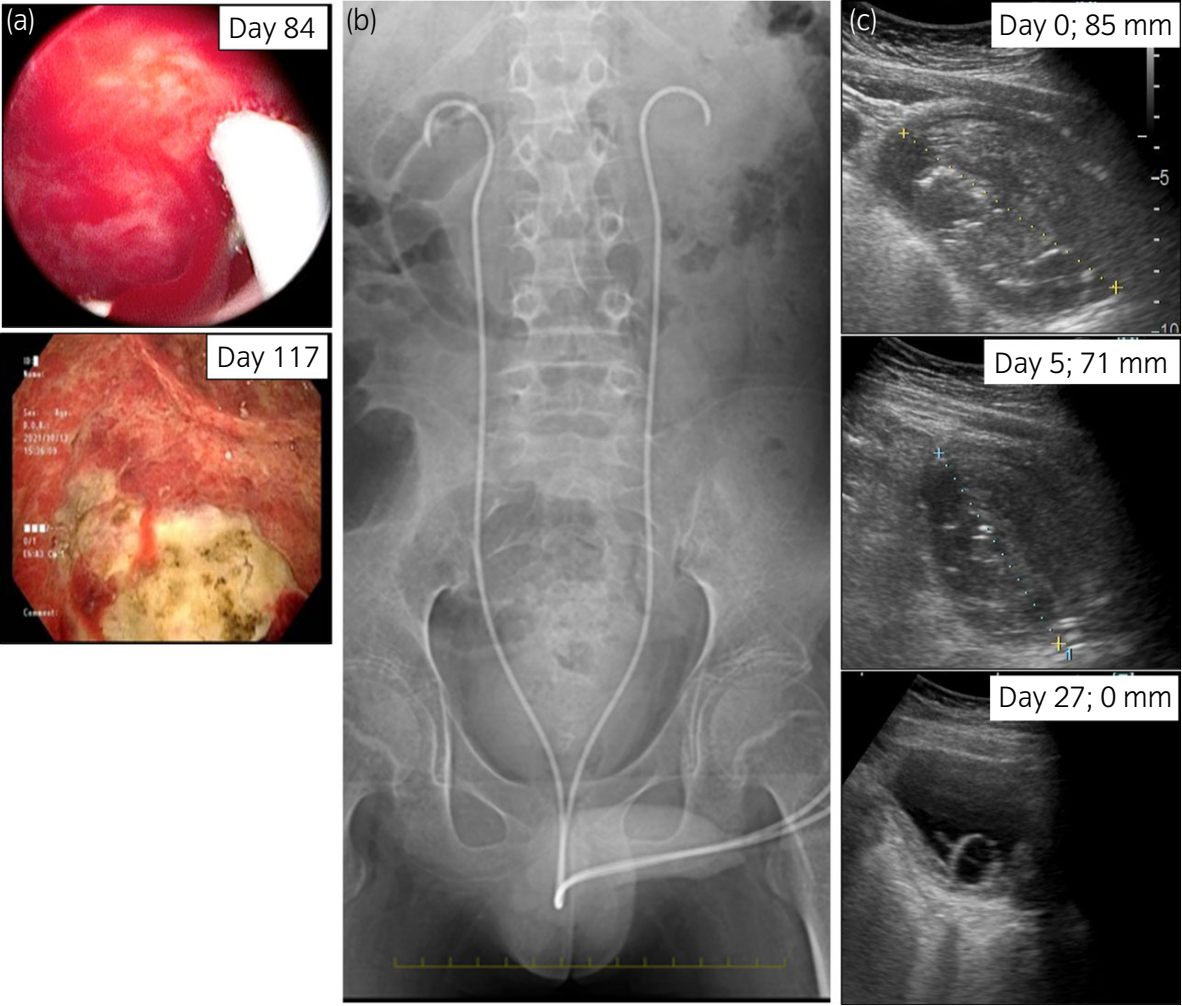
Urologic interventions and the change of the bladder blood clot. (a) Images of the bladder wall during TUE with cystoscope on day 84 (upper panel) and day 117 (lower panel) after HSCT. (b) X‐ray image taken during bilateral single‐J ureteral stent placement. (c) Sagittal section on ultrasonography of the bladder blood clot on days 0, 5, and 27 post‐stenting. The long diameter of the clot was reduced from 85 to 71 mm and then to 0 mm. [Colour figure can be viewed at wileyonlinelibrary.com]

The BKV‐HC with bladder clot retention persisted for 4 months with temporary improvement and recurrence; hence, frequent manual bladder washout and CBI were performed each time. TUE performed under general anesthesia on days 84 and 117 also failed to improve BKV‐HC. The bladder wall was diffusely edematous and hemorrhagic (Fig. 2a). A bilateral 6 Fr single‐J stent (Fig. 2b) and 8 Fr Foley catheter were placed using a flexible cystoscope without manual bladder washout on day 120. As a result, the bladder clot gradually decreased, spontaneously drained from the catheter, and completely disappeared 27 days after stenting (Fig. 2c). The patient complained of slight pain in the external urethral meatus but not in the lower abdomen. No additional procedures, including manual bladder washout, were needed. Gross hematuria did not recur after the blood clot disappeared despite Hb level and Plt count remained low. The bilateral SJ stents were removed 97 days after being placed, followed by the removal of the Foley catheter (Fig. 1). Urine tests showed decreased BK virus levels (1.0 × 10^8^ copies/mL), at 8 months post‐HSCT, BKV‐HC has not recurred.

## Discussion

Therapy for severe HC is stressful for patients because of recurrent clot retention and the necessity for bladder irrigation.^8^

Our decision to place bilateral single‐J ureteral stents was based on the following possible mechanisms: (i) decreased bladder distension by urine, thereby reducing microtrauma; (ii) protection from urinary urokinase, which prevents clot formation; (iii) bladder packing by the clot and subsequent bladder mucosal healing and bleeding cessation.^4^ Foley catheter placement played an important role in maintaining the position of the stent and discharge of fibrinolyzed blood clots. In fact, the bladder did not hyperextend painfully, possibly because of the loss of influx of urine, followed by bladder packing and hemostasis by blood clots.

Urologists do not have sufficient evidence to support the management of severe HC after HSCT. We have summarized retrospective studies and a non‐randomized control study on urologic intervention for severe HC after HSCT (Table 1). CBI after the placement of a urinary catheter may be the most common procedure for HC. It significantly reduces the mean duration of HC and hospitalization, with no adverse effects, and also reduces the incidence of late‐onset HC.^3^ However, upon defining a case in which the irrigation line was completely blocked as a failure of CBI, high CRP values (>8.89 ng/mL), low age (<14.5 years), and late‐onset time of HC after HSCT (>37 days) were reported to be independent risk factors for failure.^11^ For urinary diversion with a surgical approach, the most common procedure is bilateral PN, followed by PCy. In patients with bilateral PN, the mortality rate was 55%, similar to the total mortality rate of severe HC.^12^ Another report showed that bilateral PN was not associated with increased mortality, and no deaths were observed that were directly attributed to PN.^13^ In patients requiring PCy, the rates of mortality and transfusion requirement were significantly higher than in those who responded to medical therapy. Thus, PCy for severe HC was suggested to be undertaken only after the failure of medical therapy.^14^ Cystectomy with urinary diversion, TUE, and laser vaporization as other urinary interventions were reported only in case reports.^6^, ^8^, ^9^, ^15^ Some procedures lead to temporary or complete hemostasis; however, cystectomy, carries substantial risk in immunocompromised patients, should be considered a last resort.^4^

**Table 1:** Studies of urologic intervention for HC after HSCT

Author	Year	Type of study	Procedure	No. of patients	Outcomes
Hadjibabaie *et al*.	2008	Non‐randomized controlled study	CBI	40 (CBI) *vs* 40 (control)	CBI reduced the duration of HC
					CBI reduced the incidence of late‐onset HC
Yang *et al*.	2020	Retrospective study	CBI	227	Independent risk factors for failure of CBI were higher CRP, lower age, and late onset HC
Lukasewycz *et al*.	2012	Retrospective study	Bilateral PN	11 out of 40	45% of HC resolved within 30 days. The mortality rate was 55% in PN, same as the total mortality rate of severe HC
Au *et al*.	2017	Retrospective study	Bilateral PN	5 out of 43	PN was not associated with increased mortality. No deaths that were directly attributed to PN
Baronciani, *et al*.	1995	Retrospective study	PCy	11 out of 73	Higher mortality rate than responder of medical therapy

To our knowledge, this is the first report of successful treatment of BKV‐HC after HSCT using bilateral single‐J ureteral stenting. The mechanism of hemostasis is similar to that of PN; therefore, similar effects to PN can be expected without the risk of kidney bleeding. Our method is simple and less invasive; in addition, it does not impair the patients' activities of daily living compared to the methods in previous reports. This procedure should be attempted before surgical intervention for severe BKV‐HC after HSCT in combination with appropriate pain control. The age of the patient is an important consideration for our procedure; at the very least, patients should be old enough to undergo cystoscopy. The risk of developing BK virus‐related nephropathy due to the placement of ureteral stents should be considered^16^, ^17^ even though the patients already have BK viruria.

## Conclusion

Bilateral single‐J ureteral stenting followed by Foley catheter placement is a simple and effective method for the treatment of BKV‐HC. Hence, it should be considered before surgical interventions.

## Author Contributions

Akira Fujita: Conceptualization; data curation; visualization; writing – original draft. Kohei Kobatake: Conceptualization; writing – review and editing. Takafumi Fukushima: Investigation. Kenshiro Takemoto: Investigation. Syunsuke Miyamoto: Investigation. Hiroyuki Kitano: Validation. Kenichiro Ikeda: Methodology. Keisuke Goto: Methodology. Keisuke Hieda: Methodology. Shuhei Karakawa: Resources. Tetsutaro Hayashi: Methodology. Jun Teishima: Supervision. Nobuyuki Hinata: Supervision.

## Conflict of interest

The authors declare no conflict of interest.

## Approval of the research protocol by an Institutional Reviewer Board

Not applicable.

## Informed consent

The patient involved provided informed consent for the publication of this study.

## Registry and the Registration No. of the study/trial

Not applicable.
